# Monoaminergic Antidepressants in the Relief of Pain: Potential Therapeutic Utility of Triple Reuptake Inhibitors (TRIs)

**DOI:** 10.3390/ph4020285

**Published:** 2011-01-26

**Authors:** Guillaume Hache, François Coudore, Alain M. Gardier, Bruno P. Guiard

**Affiliations:** Faculty of Pharmacy, EA 3544, University of Paris XI, Châtenay-Malabry cedex F-92296, France

**Keywords:** antidepressant, serotonin, norepinephrine, dopamine, monoamine transporters, mood disorders, pain, SSRI, NRI, SNRI, triple reuptake inhibitors

## Abstract

Over 75% of depressed patients suffer from painful symptoms predicting a greater severity and a less favorable outcome of depression. Imaging, anatomical and functional studies have demonstrated the existence of common brain structures, neuronal pathways and neurotransmitters in depression and pain. In particular, the ascending serotonergic and noradrenergic pathways originating from the raphe nuclei and the locus coeruleus; respectively, send projections to the limbic system. Such pathways control many of the psychological functions that are disturbed in depression and in the perception of pain. On the other hand, the descending pathways, from monoaminergic nuclei to the spinal cord, are specifically implicated in the inhibition of nociception providing rationale for the use of serotonin (5-HT) and/or norepinephrine (NE) reuptake inhibitors (SSRIs, NRIs, SNRIs), in the relief of pain. Compelling evidence suggests that dopamine (DA) is also involved in the pathophysiology and treatment of depression. Indeed, recent insights have demonstrated a central role for DA in analgesia through an action at both the spinal and suprasinal levels including brain regions such as the periaqueductal grey (PAG), the thalamus, the basal ganglia and the limbic system. In this context, dopaminergic antidepressants (*i.e.*, containing dopaminergic activity), such as bupropion, nomifensine and more recently triple reuptake inhibitors (TRIs), might represent new promising therapeutic tools in the treatment of painful symptoms with depression. Nevertheless, whether the addition of the dopaminergic component produces more robust effects than single- or dual-acting agents, has yet to be demonstrated. This article reviews the main pathways regulating pain transmission in relation with the monoaminergic systems. It then focuses on the current knowledge regarding the *in vivo* pharmacological properties and mechanism of action of monoaminergic antidepressants including SSRIs, NRIs, SNRIs and TRIs. Finally, a synthesis of the preclinical studies supporting the efficacy of these antidepressants in analgesia is also addressed in order to highlight the relative contribution of 5-HT, NE and DA to nociception.

## 1. Neurobiology of Pain and Its Modulation by Monoamines

Pain is a subjective experience that results from transfer and brain analysis of various information such as the nature, location, intensity and duration of a stimulus. It also involves adaptation and modulation of the nociceptive messages by various neuromediators and related receptors. Since these neuromediators are present in the central network of brain structures that process or regulate nociceptive information, it is difficult to dissociate the affective dimension of pain from its sensory dimension [1]. Anatomically, several brain regions have been implicated in both depressive disorder and pain.

At the peripheral level, the pain comes from direct or indirect stimulation and sensitization of nociceptors by various endogenous signalling molecules, including ions, prostaglandins and/or leukotriens, histamine, bradykinines, but also monoamines [2]. The activation of nociceptors creates action potentials that are transmetted by Aδ fibers (fast-conducting, location) and/or C fibers (delayed transmission, a feeling duller and less localized) leading to a more diffuse pain. Both types of pain fibers terminate in the superficial layers of the dorsal horn of the spinal cord where several neuropeptides but also 5-HT and NE play a major role in antinociception [3,4]. Fibers from the transmission cells of substantia gelatinosa convey impulses to the thalamus, the main brain region responsible for the integration of pain input. From the thalamus, third-order neurons transmit pain impulses to the cerebral cortex where further processing occurs resulting in pain awareness [5]. In this review, particular attention will be focused on the central brain monoaminergic regions and their pathways that regulate the nociceptive information at the central level. Indeed, given the close anatomical relationship between areas involved in pain and emotion and the emotional nature of pain, it is possible that treatment of mood disorders with monoaminergic antidepressants display a powerful impact on pain by regulating the affective, emotional and sensory dimensions of pain.

### 1.1. Spinal Cord, Ascending and Descending Pathways

At the spinal level, the central terminal of the nociceptor forms synapses with neurons of the superficial dorsal horn of the spinal cord. Glutamate seems to be the main neurotransmitter released in response to a nociceptive stimulus. Glutamate then acts on post-synaptic receptors present in: (i) the projection of cells whose axons convey information to various parts of the brain; and (ii) interneurons (both excitatory and inhibitory) that all contribute to the local modulatory circuit in the spinal cord. Thus, the ascending pathways distribute spinal action potentials to brain areas related to the two dimensions of pain perception, sensory and affective: the somatosensory cortex, the periaqueductal grey (PAG), hypothalamus and basal ganglia. Spreading from central projections, corticolimbic pathways are also activated. These sites which process noxious activation include the thalamus, insular cortex, anterior and posterior cingulated cortex, prefrontal cortex [6-8] but also the amygdala [9] and the hippocampus [10]. All these brain regions are endowed with a rich serotonergic, noradrenergic and/or dopaminergic innervations suggesting the role of monoamines in the modulation of pain.

**Figure 1:**
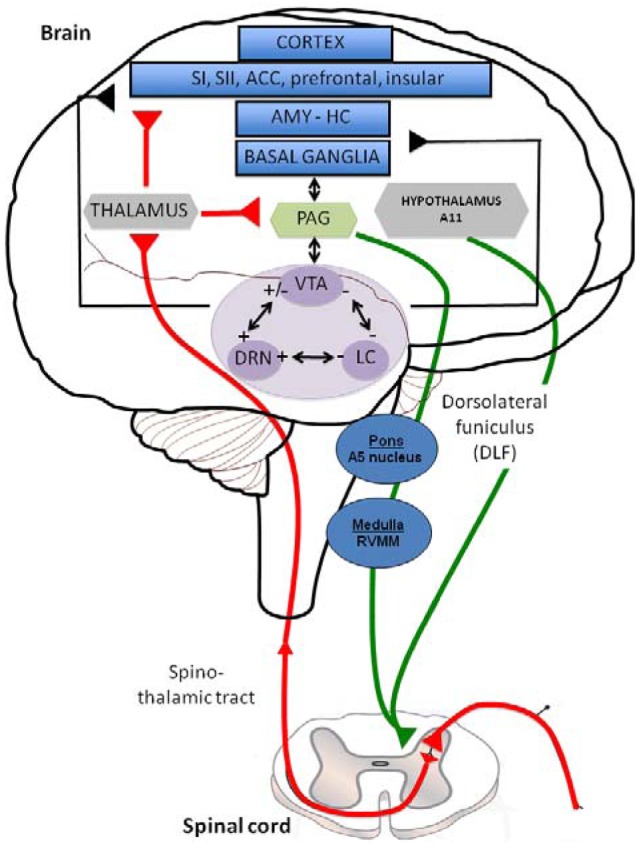
Schematic illustration of the main pathways involving monoaminergic systems in the modulation of pain perception.

Descending inhibitory or facilitatory pathways from brain areas converge at the dorsal horn, controlling peripheral inputs from nociceptors. Monoaminergic fibers originating from various brainstem nuclei control pain perception through the release of 5-HT and NE in the superficial dorsal horn via the dorsolateral funiculus (DLF) [11]. DLF fibers from descending pathway are thus comprised of serotonergic projections from the raphe nuclei and noradrenergic projections from the locus coeruleus (LC) [12]. Both 5-HT and NE contribute to the modulation of pain, constituting a gating mechanism that control impulse transmission in the dorsal horn (Figure 1). In this figure the limbic system (blue structures) includes various cortical subregions such as the somatosensory (SI and SII), anterior cingulate (ACC), prefrontal and insular cortex but also the amygdala (AMY), hippocampus (HIPP) and basal ganglia. All these structures, involved in the initiation of the descending controls of nociceptive information, are innervated by serotonergic, noradrenergic and dopaminergic neurons originating in the dorsal raphe nucleus (DRN), locus coeruleus (LC) and Ventral Tegmental Area (VTA) (purple structures); respectively. Distinct populations of monoaminergic neurons, via the dorsolateral funiculus (DLF) borrow descending pathways (green circuits) to exert a strong inhibitory effect on pain transmission in the dorsal horn (mediated by 5-HT, NE and likely DA, which may produce a local release of opioids). In particular, serotonergic inputs to the dorsal horn originate in neurons of the rotstral ventromedial medulla (RVM), including the raphe magnus and the nucleus reticularis magnocellularis. The noradrenergic innervation of the dorsal horn originates from several cell groups in the pontine tegmentum, including the A5 group. The main source of descending dopaminergic innervation of the dorsal horn is the A11 neurons of the periventricular posterior hypothalamus [13]. On the contrary, ascending pathways (red circuit) via the spino-thalamic tract, excites neurones in the periaqueductal grey matter (PAG: yellow structure) and thalamus (grey structure), which have direct and/or indirect interactions with the limbic system and monoaminergic nuclei. Interestingly, all the three monoaminergic nuclei display anatomical and functional reciprocal interactions (black arrows) regulating the release of 5-HT, NE and DA in their projections areas and thereby the sensory and emotional dimensions of pain.

Compared with the enormous literature devoted to 5-HT and NE, the spinal action of DA has received less attention. It has been proposed that the dopaminergic innervation of the spinal cord may originate in the substantia nigra and hypothalamus [14]. Further, the purported existence of a small population of DA-synthesising cells in the dorsal root has been confirmed [14]. It is also possible that NE neurons themselves constitute an important source of DA in the dorsal horn to control pain. Although this property has yet to be determined in the spinal cord, several studies reported that the clearance of DA in various brain regions, may be mediated, at least in part, by the selective NE transporter NET [15-18].

### 1.2. The Brain

The periaqueductal grey (PAG) is an important nociception modulation site where the emotional and cognitive sensations from thalamic or anterior cortical areas meet the vegetative aspects from the hypothalamus [19,20]. Although, the PAG is indirectly connected to the dorsal horn of the spinal cord through adjacent regions of the pont and the medulla [21], it initiates descending and ascending inhibition resulting in the reduction of pain. Consistent with this observation, it has been demonstrated that stimulation of the PAG produces a profound antinociception [22,23] whereas its electrolytic lesion reduced the analgesic effect of morphine [24]. These data suggest that this brain region is a major site of action of opiates in producing analgesia. However, descending facilitatory projections from the PAG to the RVM may enhance spinal nociceptive transmission of peripheral inputs.

The dorsal raphe nucleus (DRN) is interconnected and functionally related to the PAG. Although both brain regions display strong anatomical interactions [25-28], the mechanism underlying their role in the modulation of pain is not fully elucidated. The PAG modulates incoming pain information by activating DRN in the rostral ventromedial medulla, which in turn causes the 5-HT release in the dorsal spinal cord to inhibit incoming sensory stimuli [29]. It was proposed that substance P (SP), which is increased in response to a nociceptive stimulus, regulates both enkephalin and 5-HT neurotransmission in the PAG and the DRN [30]. Interestingly, a subpopulation of non-serotonergic neurons arising from the DRN could release SP in the PAG to produce a robust antinociception [31-34], particularly by evoking a release of enkephalin [35]. In turn, the PAG might also send SPergic projections to the DRN. Multiple sources of evidence suggests that SP activates serotonergic neurons in the DRN [36,37] suggesting that reciprocal interactions between the serotonergic and tachykininergic systems might be an important substrate for reducing pain.

The LC and the A7 catecholamine cell groups, known to contain spinally noradrenergic neurons, are connected to the PAG through a monosynaptic pathway [38,39]. This provides direct anatomical evidence that this pathway may mediate at least some of the effects produced by activation of neurons in the PAG. For example, activation of projection neurons in the PAG has a predominantly inhibitory effect on LC neurons [40], this action contributing to the antinociception produced by PAG stimulation. Two neurochemicals have been suggested to be involved in the modulation of LC NE neuronal activity such as corticotropin-releasing factor (CRF) and the endogenous opioid enkephalin [41]. CRF has been shown to increase the spontaneous discharge rate of LC neurons [42] whereas enkephalins exert mostly inhibitory effects on LC neurons [43]. The balance between opioids and CRF influences in the LC regulate noradrenergic transmission and likely pain through the stimulation of descending pathways.

Dopaminergic neurons from the ventral tegmental area (VTA) have no clear anatomical and direct functional interactions with the PAG. Interestingly, several studies have localized the antinociceptive effects of morphine to the PAG [24,44-46]. Together with the observation that the lesion of DA neurons by the neurotoxin 6-OHDA (injected with a norepinephrine reuptake inhibitor to prevent depletion of NE neurons), caused a decrease in the effect of morphine [24], these results suggest that an intact DA system is necessary to the antinociceptive effect of morphine, particularly in the PAG [47]. In agreement with this hypothesis, a subpopulation of neurons within the PAG is dopaminergic. These neurons project to the central nucleus of the amygdala, ventral striatum, and locally within the PAG [48,49]. Since there is substantial overlap in the neural systems containing opioid and dopamine receptors [50-52], it has been proposed that the antinociceptive effect of morphine results from the release of DA in the PAG, which in turn would facilitate the local action of opioids [53].

The thalamus is the main relay site for nociceptive inputs to cortical and subcortical structures. It includes several nociceptive nuclei of the somatosensory and intralaminar thalamus. Thalamocortical networks that produce both sensory discriminative and affective components of the pain response generate conscious pain. Initial studies have indicated that the ablation of the parafascicular nucleus (PFN) selectively reduces the emotional suffering associated with acute and chronic pain in humans, and reduces responses to noxious stimulation in animals [54].

The DRN projects axons to the thalamus including the PFN, to suppress the pain sensations. Stimulation of the DRN has been found to effectively inhibit the responses induced by noxious stimulation of neurons in the PFN [55]. In contrast, the lesion of 5-HT neurons by the specific neurotoxin 5,7-DHT abolished the effect of DRN stimulation on pain-induced excitation of the PFN. These results indicate that 5-HT has a tonic inhibitory influence on responses to noxious stimulation. It was further found that, similar to DRN stimulation itself, iontophoretic application of 5-HT in the PFN inhibits changes caused by noxious stimulation [56] suggesting that the DRN is involved in pain modulation in this ascending pathway.

The LC, but also the VTA, send projections to the somatosensory thalamus [57-59]. The involvement of ascending noradrenergic innervation of the somatosensory thalamus in pain processing is supported by a recent study showing that nociceptive stimulation activates LC neurons projecting to the thalamus [60,61]. About the role of DA, recent study reported that the local application of DA in the PFN modulates the frequencies of pain-excited and pain-inhibited neurons [62], raising the possibility that DA in this brain region play an important role in the modulation of the nociceptive response. However, it seems that DA produces dual modulatory effects depending on the DA receptor subtypes [63].

Apart from the well-known involvement in motoric circuitry of the basal ganglia, these brain nuclei are involved in many neuronal pathways having emotional, motivational, associative and cognitive functions as well. This brain region contains several nuclei including the putamen, caudate nucleus, globus pallidus, subthalamic nucleus, and nucleus accumbens that receive multimodal input from all sensory systems and thereby serve as a gating station for continuous sensory information, including pain. Several studies have suggested that basal ganglia may be involved in the sensory-discriminative aspect of pain, the affective and cognitive aspect of pain but also the modulation of nociceptive information and sensory gating of nociceptive information to higher motor areas, because they are the main link between the thalamus and the cerebral cortex [64]. Data supporting a role for the basal ganglia in pain and analgesia processing have been derived from numerous clinical and preclinical studies [65]. For example, lesions of the basal ganlgia in patients suffering from Parkinson's disease have offered further insights into the potential role of this brain region in pain and analgesia. Indeed, infarction of the lenticular nucleus (composed of the putamen and globus pallidus) may result in sensory deficits including pain in some patients [66], whereas both unilateral and bilateral deep brain stimulation of the globus pallidus have been reported to relieve pain [67]. The basal ganglia receive inputs from all cortical areas (including medial and orbital, prefrontal, dorsolateral, premotor and motor cortex, sensorimotor and parietal cortex) and the thalamus, which are endowed with a rich innervation composed of serotonergic, noradrenergic and dopaminergic nerve terminals.

The DRN heavily innervates the nucleus accumbens [68], a brain region receiving β-endorphin containing nerve terminals originating from the arcuate nucleus [69]. In humans, rats, and many other species, injection of β-endorphin into the nucleus accumbens exerts an analgesic effect [70-73]. It was hypothesized that the effect of 5-HT on chronic pain might be due to an interaction with endogenous opioid systems [74,75]. In line with this hypothesis, it has been shown that local application of 5-HT can facilitate the release of β-endorphin in the arcuate nucleus and nucleus accumbens [76]. The involvement of 5-HT in the nucleus accumbens in mediating the antinociceptive effect was further suggested by the finding that the local application of cinanserin, a 5-HT_2_ receptor antagonist, attenuated the antinociceptive effect of morphine [77]. Several possibilities has been raised regarding the interaction between 5-HT and β-endorphin: (i) the existence of enkephalins in about one third of the neurons located in the DRN [78] suggests that 5-HT and enkephalins may act as co-transmitters in the synaptic events with one playing a regulatory function for the other; (ii) 5-HT released in the nucleus accumbens may activate the enkephalinergic interneurons within the nucleus, as demonstrated in the caudate putamen [79]; (iii) enkephalins may accelerate the release of 5-HT, although no evidence is yet available in favor of this possibility.

The LC send projection to the striatum [or caudate-putamen (CPu) in human] as suggested by immunohistochemical and fluorescence histochemical studies [80]. The CPu is one of the important components of the basal ganglia, and is recognized as one of the several sites involved in the modulation of nociceptive sensory input through descending controls to the level of the spinal cord. It is rich in monoamine neurotransmitters, including NE and DA [80,81]. Neurons in the CPu respond to noxious thermal, mechanical and electrical stimulation [64,82]. Interestingly, the stimulation of the CPu induces analgesia [83] while the CPu display two types of neurons named PEN and PIN (“on-cells” and “off-cells”), which are excited or inhibited respectively by nociceptive stimulation. NE potentiated the electric activities of the evoked discharges of PEN and simultaneously attenuated those of PIN, *i.e.* exhibiting the hyperalgesic effects of NE [84]. The latter study illustrate the fact that NE is involved in the modulation of nociceptive information transmission through an action in the CPu [85].

The VTA and substantia nigra (SN) send dense projection to the nucleus accumbens and basal ganglia [86]. Clinical and behavioral data indicate that dopaminergic pathways are involved in central pain processing. Data from electrical and chemical (*i.e.*, DA receptor agonists and antagonists) stimulation or electrolytic and chemical lesions of the CPu, GP and substantia nigra (SN) provide evidence that the basal ganglia can modify behavioral responses to noxious stimulation. For example, it has been shown that a unilateral lesion of the nigrostriatal pathways causes hyperalgesic responses to painful stimuli at contralateral side [64,87-89]. As well, DA depletion by 6-OHDA injection into the medial forebrain bundle, CPu and SN results in hypersensitivity to mechanical [90,91], electrical [92] and thermal stimulation [91]. A recent study found hyperalgesic responses to painful chemical stimulation of the hind paw ipsilateral to intrastriatal 6-OHDA administration [88]. The role of DA in pain needs to be further investigated. Nevertheless, it seems that the activation of nigro-striatal dopaminergic transmission is associated with individual variations in subjective ratings of sensory and affective qualities of pain, whereas mesolimbic activation appears associated with variations in emotional responses during pain challenge [20].

The limbic system mainly consists of the anterior cingulated cortex (ACC), the insular cortex (IC), the prefrontal cortex (PFC), the amygdala and the hippocampus. Monoaminergic neurons project into these various brain regions and are involved in the regulation of pain, mood and in the affective dimension of pain [93]. Dysfunction of these ascending projections from the DRN, LC and VTA may contribute to the classical symptoms of depression. The activation of cortical structures has been shown in humans using imaging studies in response to pain [94-98]. Lesions of cortical region such as the ACC significantly reduced acute nociceptive responses [99]. The involvement of the ACC in pain modulation may be attributable to the activities of a variety of neurotransmitters as DA and glutamate [7]. Indeed, it has been reported that increased activity of glutamatergic projections intensifies nociception whereas dopaminergic projections into the ACC inhibits nociception [100]. DA also appears to be key neurotransmitters in nociception modulation in the IC that displays a high density of DA fibers arising principally from the VTA and SN [101].

The role of 5-HT and NE in these cortical regions is also well documented. For example, the increase in 5-HT extracellular levels induced by the inhibition of the 5-HT transporter (5-HTT) in the primary somatosensory cortex produces anti-hyperalgesic and anti-allodynic effects [102].

About the insular cortex, essentially considered as the anatomical substrate for integration and processing information from different functional systems, the mid-posterior part has been involved in somato and viscerosensory painful stimuli. Dense connections and interconnections between the different cortical areas allow multimodal integration of both informations [103].

The amygdala performs a primary role in the formation and storage of memories associated with emotional and affective events [104] and plays a key role in attaching emotional significance to pain [105]. Amygdala receives input from LC noradrenergic projections [19] and is involved in defense response, *i.e.* analgesia, associated with intense fear and dangerous situations [106]. Imaging studies showed an activation of the amygdala in response to different painful stimuli [107]. Changes in 5-HT receptor function in the amygdala were observed under a chronic pain-like state [102]. Apart interactions with hypothalamus and brainstem, it has been described that amygdala is involved in cognitive effects of pain through amygdala-cortical interactions. In addition, pain-related decision-making deficits involve increased GABAergic synaptic inhibition in prefrontal cortex [9].

Finally, activation of the hippocampus has been demonstrated in healthy volunteers in response to a pain stimulus [108], and preclinical studies have reported changes in the hippocampal morphology, cell proliferation and gene expression in response to chronic pain [109,110]. Since the hippocampus receives a dense monoaminergic innervation, it is possible that the increase in extracellular levels of 5-HT, NE and DA, each monoamine known to stimulate neurogenesis and the expression of neurotrophic factors in the hippocampus [111-113], may produce antinociceptive effects. This is in agreement with a recent study showing that chronic pain suppresses the increase in the immunoreactivity of doublecortin-positive cells (a marker of neuron maturation) induced by an enriched environment [114].

## 2. Pharmacological Properties of Monoamines Reuptake Inhibitors

For many years, studies mainly focused on the serotonergic and the noradrenergic systems because of the efficacy of selective 5-HT or NE reuptake inhibitors (SSRIs/NRIs) in the treatment of major depressive disorder. SSRIs and NRIs block the 5-HT or NE transporter (5-HTT or NET), respectively; thereby increasing extracellular concentrations of these monoamines in the synapse and prolonging their duration of action at postsynaptic level. Despite the variety of SSRIs (citalopram, escitalopram, fluovoxamine, fluoxetine, paroxetine and sertraline) and NRIs (atomoxetine, desipramine, reboxetine), their binding property towards monoamine transporters may vary [115]. In addition, since close anatomical and functional interactions between monoaminergic systems exist, any action on one system may reverberate in the other system [116]. A corollary of this cross-modulation is that the *in vivo* net effect of SSRIs or NRIs on 5-HT or NE neurotransmission is difficult to anticipate. Functional *in vitro* and *in vivo* approaches have thus been applied to characterize the pharmacological properties of these antidepressants. Inhibition of [^3^H]-5-HT or [^3^H]-NE reuptake in synaptosomes, is one of the most widespread method to assess the *in vitro* potency of reuptake inhibitors [117] and to predict indirectly, their affinity and selectivity on biogenic amines transporters. Intracerebral electrophysiology and microdialysis have proven to be sensitive methods to assess the *in vivo* inhibitory potency of various drugs on reuptake. Indeed, at presynaptic level, when 5-HTT or NET are blocked on the serotonergic or noradrenergic cell bodies, respectively, there results an accumulation of 5-HT or NE in the vicinity of somatodendritic 5-HT_1A_ or α_2_ autoreceptors in the dorsal raphe (DR) or locus coeruleus (LC). This lead to an attenuating firing DR 5-HT and LC NE neurons in a dose-dependent manner due to the activation of these neuronal elements exerting a negative feedback influence [118]. This parameter can be used to characterize the pharmacological profile of reuptake inhibitors. At nerve terminals, an accumulation of 5-HT or NE also occurs in response to the inactivation of the 5-HTT or the NET by SSRIs or NRIs, and the enhancement of extracellular levels of monoamines can be probed by microdialysis in various brain regions [119] and constitutes another parameter to study the functional activity of reuptake blockers. Nevertheless, since microdialysis methodology may vary between laboratories, the electrophysiological approach seems to be the most appropriate approach to establish relevant comparisons between compounds.

### 2.1. Single- and Dual-Acting Monoamine Reuptake Inhibitors

The six approved SSRIs in the treatment of depression are all potent 5-HT reuptake inhibitors *in vitro* as well as *in vivo* [117,120]. Paroxetine is the most potent inhibitor of 5-HT transporter, whereas citalopram and escitalopram are the most selective ones [115,117]. However, the 5-HT/NE ratios vary considerably between SSRIs (Table 1). Multiple sources of evidence from electrophysiological studies indicate that SSRIs reduce the firing activity of DR 5-HT neurons in rats with ED_50_ ranging between (60 and 600 μg/kg; i.v.). It is important to note that some of them also produce a weak, but significant decrease in LC NE and VTA DA neuronal activities after acute or sub-chronic administration (Table 2). As expected, microdialysis studies showed that all SSRIs enhance the extracellular levels of 5-HT. Interestingly, SSRIs may also enhance the extracellular levels of NE in the frontal cortex and hippocampus after acute or chronic administration in rodents [15,121-129]. Different hypotheses have been raised to explain such effects of 5-HT reuptake inhibitors on the noradrenergic system. It is possible that an increase in 5-HT is a prerequiste to stimulate the central NE transmission. Accordingly, the release of NE in serotonergic nerve terminals areas was observed in response to the activation of postsynaptic 5-HT_1A_ [130] or 5-HT_3_ receptor types [131]. Together with the observation that SSRIs decreased the spontaneous neuronal activity of NE neurons in LC [132-134], this suggests that the enhancing property of SSRIs on the noradrenergic system may involve a local excitatory mechanism at nerve terminals, independent from the inhibition of the NET. Given the high degree of homology between monoamine transporters, another possibility would be that SSRIs exert non-selective effect through the blockade of the NET. In line with this assumption it was shown that paroxetine blocks the NET [135,136]. In addition, we have recently shown that the enhancement of cortical extracellular levels of NE induced by escitalopram remained intact in 5-HTT^−/−^ mice, while the increase in cortical extracellular levels of 5-HT was suppressed (unsubmitted data), confirming the possibility that SSRIs may also inhibit, at least partially, the NET. Aberrant uptake of NE from serotonergic nerve terminals might also explain that SSRIs increase the extracellular levels of NE [137] but this latter point is still debate of matter. Finally, the pharmacological profile of SSRIs and more paticularly their monoaminergic receptor-profile may also play a significant role in the modulation of NE neurotransmission. As an example, the 5-HT_2C_ blocking activity of fluoxetine [138] may prevent SSRI-induced decrease in LC NE neuronal activity [139] and consequently may participate in stimulating NE at nerve terminals.

Atomoxetine, desipramine and reboxetine are potent and selective NET inhibitors [150]. Their capacity to inhibit the NET *in vitro* is in the nanomolar range, whereas data from synaptosomes studies indicate that their potency to inhibit 5-HT reuptake is at least 50 times weaker (Table 1). In term of electrophysiological property, desipramine and reboxetine inhibit the firing activity of rats LC NE neurons with an ED_50_ of 110 and 480 μg/kg (i.v.), respectively. Accordingly, *in vivo* microdialysis experiments have revealed that this class of antidepressants is very potent at enhancing brain NE transmission in the rat frontal cortex, hippocampus and nucleus accumbens either after acute or chronic administration [15,151-156]. In contrast, these drugs had no effect on the DR 5-HT and VTA DA neuronal activities (Table 2), confirming their selectivity towards the NET. Nevertheless, the effects of reboxetine on the serotonergic system remains equivocal since an inhibition [157] or an increase [144,158] in 5-HT firing activity in rats was described after its systemic administration. Moreover, although additional works are required to elucidate the impact of NRIs on the serotonergic system, some studies failed to demonstrate that reboxetine increased 5-HT outflow [156,159]. Svensson and colleagues found that in rats it enhanced the extracellular levels of 5-HT despite their apparent low affinity for 5-HT reuptake sites [158]. Hence, NRIs may cause a secondary enhancement of central serotonergic activity by a mechanism separate from 5-HT reuptake inhibition. Accordingly, it has been proposed that the enhanced levels of synaptic NE in postynaptic regions likely enhanced serotonergic activity through the stimulation of postsynaptic α_1_-adrenoceptors located on 5-HT cell bodies in the DR [160,161]. Interestingly, since prolonged administration of desipramine or reboxetine desensitize α_2_-adrenergic receptors present on 5-HT terminals, it is possible that such a mechanism lead to an increase in synaptic availability of endogenous 5-HT as observed in the rat hippocampus [158].

A second generation of antidepressants targeting both 5-HT and NE, named serotonin and norepinephrine reuptake inhibitors (SNRIs), has then been developed with the hope to produce more robust therapeutic effects in depression. Among these antidepressants, duloxetine, milnacipran and venlafaxine have provided one of the first opportunities to propose that a specific dual-acting antidepressant would be significantly more effective than SSRIs in depressed patients [174,175]. Indeed, it is currently believed that the therapeutic efficacy of antidepressant drugs may depend on their capacity to enhance simultaneously brain 5-HT and NE neurotransmissions. Although SNRIs inhibit both 5-HTT and NET, there are considerable differences in their affinity, selectivity and potency [115] (Tables 1). *In vitro,* duloxetine and venlafaxine have a high affinity for the 5-HTT compared to the NET whereas milnacipran has a more balanced affinity for these transporters (Table 1). Electrophysiological experiments in rats have confirmed these pharmacological properties since duloxetine and venlafaxine reduce the firing activity of DR 5-HT neurons with an ED_50_ lower than that obtained for LC NE neurons (Table 2). Surprisingly, although high doses of milnacipran are required to inhibit the discharge of 5-HT neurons in rats, it does not modify the neuronal activity of NE neurons (Table 2). The effects of the three SNRIs on the extracellular levels of 5-HT and NE have been extensively studied. In freely moving rats duloxetine produced a dose-dependent increase in the extracellular levels of both 5-HT and NE in the frontal cortex or hypothalamus [176-178]. Venlafaxine enhanced the levels of NE but not 5-HT in the cortex [179,180]. Other studies, however, found a dose-dependent increased of both 5-HT and NE concentrations in the frontal cortex and hippocampus [124,144,181,182] with a greater increase of 5-HT output compared with that of NE [122]. About milnacipran, microdialysis studies revealed that it produced a dose-related similar increased in both extracellular 5-HT and NE in the guinea pig hypothalamus [141,183] in agreement with its *in vitro* pharmacological profile [184]. It is interesting to note that, in agreement with their pharmacological profile, neither venlafaxine nor milnacipran increased the extracellular level of DA in the rat hippocampus or frontal cortex [182,185]. However, a microdialysis study reported an enhancement of extracellular level of DA in the rat frontal cortex and nucleus accumbens in response to the administration of duloxetine [178]. These neurochemical data are surprising but agree with the observation that duloxetine displays a higher affinity for the DA transporter (DAT) [186,187], albeit weak, than the two other SNRIs.

The recent observation that pure dopaminergic drugs, such as the D_2_/D_3_ receptor agonist pramipexole, a drug without apparent affinity for either NE or 5-HT neuronal elements, are effective antidepressants [188], suggested that enhancing DA function may underlie, at least in part, a therapeutic response in major depressive disorder. So far, no selective DA reuptake inhibitors are available in clinic. Bupropion and nomifensine remain the sole drug used in the treatment of depression and sharing the property of inhibiting the DAT [118]. Nevertheless, both agents are not selective and display a noradrenergic component. Bupropion is an effective antidepressant when used alone or in combination with SSRIs that would exert its therapeutic effect through the selective blockade of the DA, but also NE transporter (Table 1). Despite this pharmacological profile, acute administration of bupropion had no effect on the firing activity of VTA DA and LC NE neuronal activities (Table 2). However, it has been shown to enhance extracellular levels of DA in the striatum and frontal cortex of mice and rats [189-192]. Together, these data indicate that bupropion display an original mechanism of action. In line with this hypothesis, positron emission tomography (PET) scan studies reported that clinically effective doses of bupropion produce very low occupancy of DA reuptake sites [193]. In preclinical studies, bupropion was hypothesized to be a NE releaser in LC and at the level of NE terminals in the DR [171]. Hence, administration of sub-acute bupropion dose increases firing of 5-HT neurons whereas NRIs do not. This effect was no longer present in NE-lesioned rats suggesting the involvement of the noradrenergic system in its mechanism of action. It is thus possible that the effect of bupropion on the serotonergic system would be mediated via α_1_-adrenoceptor, which exerts an excitatory action on 5-HT neurons activity [171,194-196].

Nomifensine is an antidepressant with potent NE and DA reuptake inhibiting properties (Table 1). Animal studies showed that an acute administration of nomifensine had no effect on the neuronal activity in the LC but it markedly decreased the firing rate of NE neurons after 2 days of treatment [172] as previously found with the NRI reboxetine. Similar to NE neurons, the firing rate of DA neurons in the VTA was significantly decreased with a short-term nomifensine regimen (Table 2) while increasing the firing rate of 5-HT neurons when the treatment was prolonged [172]. It is well documented that increases in synaptically available NE and DA, a property that was observed by using microdialysis in response to the administration of nomifensine [197]. Such neurochemical effects could also account for the activation of 5-HT neurons via excitatory α_1_-adrenoceptors [160] and D_2_ receptors [198-200] in the DR.

In summary, a wide range of monoaminergic antidepressants is thus available for clinicians. These agents with either selective or dual actions have distinct mechanisms of action and it seems important to take into consideration the high degree of connectivity between monoaminergic systems [173] to predict their *in vivo* effects. Understanding the mode of action of drugs targeting these catecholaminergic neurotransmitters can improve their utilization in monotherapy and in combination with other compounds particularly the SSRIs. Due to the purported role of DA in the depression, triple reuptake inhibitors (TRIs) could be the next generation of antidepressants after SSRIs, NRIs, SNRIs, in the treatment of depressive disorders and other symptoms such as sexual dysfunction and/or chronic pain [115].

### 2.2. Pharmacological Properties of Triple Monoamines Reuptake Inhibitors

A number of compounds with the ability to bind and block all three monoamine transporters have been developed. To illustrate the growing interest for TRIs, it is interesting to note that just in 2010, the pharmacological profiles of seven compounds have been reported [201-207]. Experiments from rat cortical synaptosomal fractions indicate that the inhibition of monoaminergic transporters by TRIs is lower than that of single- or dual-acting agents (Table 1). This indicates that the novelty of this new class of antidepressant lies in their balanced profile at blocking 5-HTT, NET and DAT rather than in their *in vitro* potency. In an attempt to further characterize the functional activity of TRIs, recent electrophysiological and neurochemical studies have been conducted. In agreement with a low *in vivo* potency in comparison with single- or dual-reuptake inhibitors, relative high intraveinous doses of the TRIs SEP225289 and DOV216303 were required to inhibit the electrical activities of DR 5-HT, LC NE and VTA DA neurons. Although this may result from a lower affinity for the monoaminergic transporters than selective reupake inhibitors [115] or from a poor brain penetration, 5 mg/kg of DOV216303, produced an inhibition of 80% of LC NE neuronal activity but only of 30% and 40% of DR 5-HT and VTA DA neurons; respectively [173]. The observation that both TRIs exerted a predominant effect in the LC, while producing only a partial decrease in DR 5-HT firing activity was puzzling given the equal *in vitro* affinity and potency of the former drugs for all three transporters. The reciprocal interactions between monoaminergic neurons might have thus contributed to alter the functional *in vivo* activity of TRIs because the majority of SSRIs, NRIs and SNRIs produce a complete suppression of DR 5-HT neurons firing (Table 2). The possibility has been raised that the lesser than expected effect of SEP225289 or DOV216303 on the firing activity of 5-HT neurons resulted, at least in part, from the accumulation of DA and NE in the DR, which are supposed to be excitatory [160,198-200]. In line with this hypothesis, when SEP225289 was administered to rats following an acute intravenous administration of the 5-HT_1A_ receptor antagonist WAY100635, the discharge of 5-HT neurons was blocked [173]. These results further demonstrate that the excitatory influence of catecholamines (*i.e.*, NE and DA) specifically unveiled in response to 5-HT_1A_ autoreceptors inactivation. The observation that bupropion or nomifensine reversed escitalopram-induced decrease in 5-HT neuronal activity [170,172] further supports the idea that the simultaneous increase in cathecholamines may counteract the electrophysiological effect of 5-HTT inhibition in the DR. Despite these results, in two recent microdialysis studies in rats DOV216303 (20–60 mg/kg) simultaneously increased all three monoamines but did not produce a greater increase in cortical 5-HT outflow in comparison to catecholamines [201,208]. It is noteworthy that JAZ-IV-22, a new TRI displaying a balanced profile close to that reported for DOV216303, produced similar neurochemical effects than the former compound [201]. PRC200-SS (5 and 10 mg/kg; i.p.) also stimulated the cortical extracellular levels of all three monoamines [149] with a marked effect on NE and 5-HT transmissions, in agreement with its *in vitro* pharmacological profile. Nevertheless, PRC200-SS failed to modify the extracellular levels of DA in the mPFC. This is somewhat surprising given the dense dopaminergic innervation and the high expression of DAT in this brain region in rats [209]. These neurochemical observations also contrast with the fact that catecholamine uptake blockers such as nomifensine, desipramine and reboxetine increased, although moderately, DA levels [210-214]. Interestingly, in the core of the nucleus accumbens (Nacc), PRC200-SS (10 mg/kg; i.p.) increased DA, and, to a lower extent, 5-HT outflow without affecting NE, probably due to the highest density of dopaminergic nerve terminals in this brain region. As a last example to emphasize the contrast between *in vitro* and *in vivo* functional activity of TRIs, JNJ7925476 produced a robust and dose-dependent increase in all three monoamines, with a maximal effect for DA (15-fold above basal level) compared to 5-HT and NE (5–7 fold above basal level) at the highest dose tested (10 mg/kg; s.c.) in the cortex of freely moving rats [215]. However, data indicate that JNJ7925476 displayed a better *in vitro* binding affinity and blocking activity for 5-HTT and NET than for DAT in rats [215]. Differences in transporter occupancy cannot explain these findings since this parameter followed the same trend observed with cortical extracellular monoamines levels. It was therefore proposed that the high cortical levels of DA might have resulted from the blockade of the NET by this drug, which displays a high affinity for the DAT [16,216]. Finally, it is interesting to note that among the recently synthesized TRIs, despite their overall balanced profile, their systemic administration in rodents resulted in considerable variations between 5-HT, NE and DA extracellular levels [205,206].

All these findings illustrate the fact that the *in vivo* activity of reuptake inhibitors will not necessarily reflect their *in vitro* functional activity, probably due in part, to the functional interactions between monoaminergic neurons. Despite such differences, all TRIs, depending on the brain region, are able to enhance monoaminergic neurotransmission to produce antidepressant-like effect in naïve or depressive-like animals. Thus, in generalized pain states in which mood changes and diffuse pain occur in relation with an attenuation of brain monoaminergic transmission, TRIs could modulate nociceptive systems and display a major role in its relief.

## 3. Monoamines Reuptake Inhibitors and Pain Preclinical Outcomes

Acute and chronic pains may in part, result from reduced levels of endogenous 5-HT, NE and DA activity, at both the spinal and supraspinal levels [217]. As described in the first part of this review and depicted in Figure 1, nociception is a bi-directional process of ascending and descending neuronal pathways involving monoaminergic systems whose activation may have an inhibitory influence on pain. Consequentially, it is presumed that 5-HT, NE and/or DA reuptake inhibitors may attenuate pain by preventing their presynaptic reuptake, leading to increased postsynaptic monoamines' levels and sustained activation of the descending pain inhibitory pathways [218]. In this part, we will describe the preclinical data supporting the role of currently available antidepressant drugs in the control of pain. Despite the complexity of pharmacological interactions between monoaminergic neurons that sometimes may attenuate monoaminergic neurotransmission (Figure 1), one would expect a better efficacy of dual-or triple-acting agents over selective 5-HT or NE reuptake inhibitors. Indeed, since all three monoamines are involved in antinociception, the recruitment of more than one system may produce beneficial effects. Although findings indicate that the antinociceptive potency of reuptake inhibitors varies according to their monoamine specificity, the nature of stimuli and the animal models of pain [219], this section may help determine the optimal choice of monoaminergic activity in the management of pain. In particular, it is important to consider the potency of monoaminergic reuptake inhibitors in animal models of pain in regard to their *in vivo* pharmacological properties towards SERT, NET and DAT and the reciprocal interactions between monoaminergic systems.

### 3.1. Serotonin Selective Reuptake Inhibitors (SSRIs)

The idea that the inactivation of SERT and the related increase in extracellular levels of 5-HT [220] could be a relevant strategy in the relief of pain, is supported by the observation that morphine-induced analgesia is potentiated in 5-HTT deficient mice [221]. Although the spontaneous pain sensitivity is unaltered in these mutant mice compared to their wild-type littermates [221], multiple sources of evidence suggest that the pharmacological blockade of 5-HTT induced by SSRIs reduces acute pain in the hotplate and tail flick tests (Table 3). For example, citalopram produces antinociceptive effects in both rats [222-224] and mice [219,225]. Such antinociceptive behaviors were reported with other SSRIs such as fluoxetine [226,227], fluvoxamine [219,222,228], paroxetine and sertraline [229-232] (Table 3). Interestingly, the effects of fluoxetine are completely blunted in 5-HT depleted animals [227,233] suggesting that SSRIs-induced antinociception involves serotonergic pathways. In addition, fluoxetine but also fluvoxamine, paroxetine and sertraline significantly potentiated the analgesic effect of morphine [234-239] (Table 3). Although these effects were blocked by naloxone, fluoxetine did not alter the binding of [^3^H]-naloxone demonstrating the lack of affinity of this SSRI for opioid receptors [239]. The site of action of SSRIs remains poorly studied and somewhat equivocal. It was reported that the effects of SSRIs may involve supraspinal structures since the intracerebroventrical, but not intrathecal, injection of citalopram mimics the effect of its systemic administration [223]. Nevertheless, this contrasts with studies demonstrating that citalopram attenuated evoked glutamate release in the dorsal horn of the anesthetized rat [240] or studies reporting that the antinociceptive effects of SSRIs resulted from an increase in opioid transmission in the spinal cord [241,242].

It is thus possible that the antinociceptive effects of SSRIs in acute pain involves both spinal and supraspinal mechanisms. This hypothesis is supported by the fact that in “positive” studies comparing the effects of SSRIs in the tail flick and hot plate tests, these pharmacological agents produced analgesic responses in both tests (Table 3). Indeed, tail flick is known to result from a spinal reflex [243] whereas in the hot plate test, licking or jumping responses, are known to be the result of supraspinal sensory integration [244,245]. In a recent study comparing the effect of various SSRIs in the hotplate test, differences between citalopram, escitalopram, fluvoxamine and fluoxetine (all SSRIs administered intraperitoneally) were yielded [225]. There are several hypotheses that may explain such differences in the antinociceptive action of SSRIs. Since all three monoamines are involved in antinociception, it is possible that the differential interaction of SSRIs with the noradrenergic and/or the dopaminergic systems may mediate an additional effect. Another possibility may arise from the differential interaction of each SSRI with monoaminergic or non-monoaminergic receptors (e.g. 5-HT_2C_ receptor for fluoxetine, sigma opioid receptor for fluvoxamine).

In chronic neuropathic and inflammatory models of pain, citalopram reduced hyperalgesia [247]. The observation that escitalopram produced more robust antinociceptive effects than citalopram [225], is in agreement with previous study showing that the R-enantiomer may antagonize and thus attenuate the effects of the active enantiomer [116,321,322]. All six SSRIs share this analgesic property in chronic models of pain in rodents since fluoxetine, paroxetine and sertraline also produced anti-hyperalgesic and/or anti-allodynic effects in streptozotocin (STZ)-induced diabetic neuropathy, sciatic nerve ligation (SNL) [241,251,254,255,257,266,268,323] or in inflammatory conditions such as formalin or acetic-acid injection [222,233,260,261,265,324] (Table 3). Surprisingly, the antinociceptive effects in chronic models of pain have been revealed after single administration of SSRIs. Despite all these positive studies, some studies failed to unveil antinociceptive effects of SSRIs in chronic pain [241,258,262,264,271]. This inconsistency of effects needs to be validated following chronic SSRIs treatments.

### 3.2. Norepinephrine Reuptake Inhibitors (NRIs)

Genetically modified mice lacking the NE transporter exhibit normal nociceptive threshold [325]. This is consistent with findings showing that two NRIs, desipramine and reboxetine, failed to modify the response to a thermal nociceptive stimulation in the hotplate test [273,278,326] (Table 3). Nevertheless, some studies have reported a weak analgesic effect of NRIs in the tail flick and hotplate tests [219,280,281,283]. This could be attributable to the systemic administration of the drugs. Indeed, it was reported that intrathecal injection of desipramine produced antinociceptive effect in the tail-flick [277], but also in other analgesic tests [284,327]. It is also possible that a prolonged administration of NRIs is required to produce antinociception. In line with this hypothesis, repeated administration of desipramine produced analgesia in the tail flick and hotplate tests [282] and potentiated the analgesic effect of morphine [277,328,329]. Another hypothesis would be that NRIs acted at the spinal level since the majority of studies on acute pain suggests that NRIs produce analgesic responses specifically in the tail flick test (Table 3).

In various models of neuropathic and inflammatory pains, both desipramine and reboxetine displayed robust antinociceptive effects by reversing allodynia and/or hyperalgesia either after acute or chronic administration in mice [256,272] and rats [262,274-276,279]. The observation that these antinociceptive behaviors were reversed by naloxone [279] strongly suggests the involvement of the opioid system in the analgesic effect of NRIs. Two others compounds, displaying a high selectivity for the NET were tested in chronic neuropathic pain in rat and appear to be promising candidate for development as novel analgesic drugs. The intrathecal administration of the conopeptide Xen2174 resulted in a long duration anti-allodynic responses in rats with chronic constriction injury (CCI) of the sciatic nerve or an L5/L6 spinal nerve injury [330] whereas WAY-318068 was shown to be efficacious in models of acute, visceral, inflammatory, neuropathic, diabetic and bone cancer pain [331].

Although the increase in extracellular levels of 5-HT or NE induced by SSRIs or NRIs seems to participate, at least in part, in attenuating pain, the impact of both monoamines to nociception remains difficult to evaluate. In a recent study, the relative contribution of 5-HT and NE in the spinal nerve ligation (SNL) model of neuropathic pain has been examined. It was demonstrated that NRIs, desipramine and reboxetine, reversed allodynia, while the SSRI fluoxetine displayed a minimal activity suggesting that compounds with greater affinity for the NET are more effective in attenuating pain than compounds with a greater affinity for the SERT [256]. Such results could be explained by the serotonin's propensity to both facilitate and inhibit pain in contrast to NE, which is purely antinociceptive. Interestingly, in the latter study, paroxetine produced similar anti-allodynic effect to that observed with NRIs. This unexpected property of paroxetine, which is reported to be a selective SRI *in vitro*, may involve the noradrenergic property of paroxetine compared to fluoxetine (Table 1). This is in agreement with previous studies showing the capacity of paroxetine to block NET [135,136] and increase the extracellular levels of NE in the frontal cortex and hippocampus of rodents [118-126]. Another study comparing the antinociceptive effects of citalopram and duloxetine showed that in the hot plate test, duloxetine significantly increased the nociceptive response latency, whereas citalopram was ineffective [247], further supporting the fact that the simultaneous enhancement of 5-HT and NE neurotransmissions may induce synergistic effects [218]. SNRIs may thus produce more robust effect than SSRIs or NRIs in animal models of pain. Nevertheless, the efficacy of the highly selective NET inhibitors Xen2174 and WAY-318068 suggests that NRI activity alone was sufficient to produce analgesia.

### 3.3. Serotonin Norepinephrine Reuptake Inhibitors (SNRIs)

As observed with NRIs, duloxetine has no or modest effect in acute models of pain in mice [289] or rats [247,293]. Interestingly, when both tail flick and hot plate were tested in the same study, antinociceptive responses were only observed in the hot plate paradigm, suggesting an involvement of supraspinal mechanism. However, it clearly appears that in both species, duloxetine produced antinociceptive effects by reducing hyperalgesia and/or allodynia in chronic neuropathic [285,293] or inflammatory models of pain [247,289,293] (Table 3). Interestingly, duloxetine has a more pronounced antinociceptive potency than milnacipran or venlafaxine [290,293]. This may result, at least in part, from the fact that duloxetine, but not milnacipran and venlafaxine, increased DA transmission [178,182,185] in addition to their neurochemical effects on the serotonergic and noradrenergic systems. This is in agreement with the pharmacological profile of duloxetine, which is more potent at blocking SERT and/or NET than milnacipran or venlafaxine (Table 1 and Table 2). Milnacipran also displays robust anti-hyperalgesic and anti-allodynic effects in various models of chronic pain such as the ligature of the sciatic nerve or the fifth lumbar nerve [102,241,271,296,299,301], or inflammatory pain [228,290,300]. The demonstration of the contribution of both serotonergic and noradrenergic systems in the antinociceptive property of milnacipran or venlafaxine comes from a recent study showing that the effects of these SNRIs can be prevented by the inhibitor of 5-HT or NE synthesis, parachlorophenylalanine or alpha-methyl-para-tyrosine; respectively [301,332]. The observation that naloxone also attenuated the antinociceptive effect of milnacipran in these models [301], emphasizes the importance of the opioid system in its behavioral effects. Although the site of action of milnacipran has yet to be determined, evidence suggests that it produces antinociceptive effect not only at the spinal but also at the supraspinal level. Indeed, the effects of the systemic administration of milnacipran were reproduced after its intrathecal administration [241,271,298]. At the supraspinal level, milnacipran attenuates the increase in c-fos expression in the ACC in response to noxious stimulation [299]. Surprisingly, in contrast to duloxetine and milnacipran, venlafaxine produced antinociceptive effects in mice and rats in acute pain assessed in the hotplate test [303,304,311]. Although venlafaxine has a weak affinity for 5-HT and NE transporters in comparison with duloxetine and milnacipran, the reasons of such difference remain obscure. It is possible that the metabolism of these drugs play an important role in their pharmacological activity. Indeed, the fact that venlafaxine is metabolized into desmethylvenlafaxine may potentiate its antinociceptive property. In rats, it has been shown that venlafaxine may act as an SSRI at low doses (<10 mg/kg; i.p.) and begin to exhibit dual reuptake activity at doses >30 mg/kg; i.p. Consequently, it was proposed that the maximal antinociceptive effect of venlafaxine was observed when it was acting as a dual re-uptake inhibitor. Finally, as observed with duloxetine and milnacipran, venlafaxine attenuated hyperalgesia and/or allodynia in various models of neuropathic [265,272,309,310,315,332,333] or inflammatory pain [307,313] (Table 3). In extension of these findings, venlafaxine dose-dependently attenuated formalin-induced nociceptive behaviors [265], an effect antagonized by naloxone [306].

Thus, although limited, studies comparing the analgesic effect of monoamines reuptake inhibitors in rodents suggest that dual acting antidepressants are more active in alleviating acute or chronic pain than selective NE reuptake inhibitors, which themselves appear more potent than SSRIs [334]. The rank of order of analgesic effects of these antidepressants is thus: SNRIs > NRIs > SSRIs.

### 3.4. Norepinephrine and Dopamine Reuptake Inhibitors

The role of DA reuptake inhibitor has been poorly studied in animal models of pain. This is likely due to the lack of selective DA reuptake inhibitors currently available. However, it was reported that the DA reuptake inhibitor, GBR12909 did not affect nociception in thermal or mechanical tests [335,336] but decreased buspirone-induced analgesia in the thermal test [336]. It was also shown that microinjection of the DA reuptake inhibitor GBR12935 into the rostral agranular insular cortex increased pain thresholds to radiant heat at baseline and produced analgesia to sustained pain from subsequent intraplanar formalin [337].

The contribution of DA and the putative interest of DA reuptake inhibitors in relieving pain must be estimated also from the behavioral effects of agents displaying a blocking DAT activity such as bupropion or nomifensine. In rats, bupropion decreased allodynia induced by CCI and spinal nerve ligation models of neuropathic pain [265]. The inability of reboxetine to reverse mechanical allodynia, in combination with *in vitro* and *in vivo* pharmacological property demonstrates that bupropion is a much weaker inhibitor of NE reuptake than reboxetine [338-340] (Table 1 and Table 2), might suggest that inhibition of DA reuptake accounted for the anti-allodynic action of bupropion. Correspondingly, the inability of bupropion, in contrast to reboxetine, to attenuate formalin-induced nociceptive behaviors and thermal hyperalgesia in CCI rats might similarly be explained by its weak potency at inhibiting the NET in contrast to reboxetine (Table 1 and Table 2). The analgesic properties of the catecholamine uptake inhibitor nomifensine were investigated in the tail immersion, hot plate and formalin tests. Systemic administration of nomifensine produced analgesia only in the formalin test. The analgesia was not affected by the opioid receptor antagonist naltrexone suggesting that nomifensine analgesia appears to be DA-mediated but independent of opioid mechanisms [316]. Nevertheless, these data contrast with previous studies indicating that nomifensine did not affect nociceptive behavior [317] (or even produced hyperalgesic effects [318].

This poor literature contrasts with extensive data showing the role of DA and related receptors in pain [341]. For example, the systemic administration of the mixed dopamine agonist apomorphine in rodents induced a biphasic dose-response characterized by hyperalgesia at low doses and analgesia at higher doses [342]. Since the antinociceptive effects of higher doses of apomorphine were antagonized by central-acting sulpiride, but not by the peripheral D2-like receptor antagonist domperidone, it was proposed that the activation of central D2-like receptor was involved in the antinociceptive effects of apomorphine. The hyperalgesia due to low dose of apomorphine could stem from selective activation of high-affinity presynaptic autoreceptors, which would attenuate normal dopaminergic reactivity in response to stimulation, whereas higher doses would be necessary to bind postsynaptic targets adequately to affect analgesia [342]. Although the central region(s) mediating the effects of DA on nociception are not clearly identified, pharmacological experiments strongly suggested that the striatum, cerebral cortex and PAG play a major role. Indeed, microinjection of the D2-like receptor agonist quinpirole within the nucleus accumbens has been found to inhibit the persistent phase of formalin-induced nociception in a dose dependent fashion while the D1-like receptor agonist SKF-38393 was without effect [343]. Nevertheless, these results do not necessarily mean that the impact of D1-like receptor in the control of pain is insignifiant. Accordingly, microinjection of a D1-like receptor antagonist into the PAG likewise attenuated opiate-induced analgesia in the hot plate test in a dose-dependent manner, while D2-like receptor antagonism was without effect. These results demonstrated that the dopaminergic network of the PAG participates in supraspinal nociceptive responses after opiate administration through the involvement of D1-like receptors [344,345]. Finally, with respect to the role of DA an related receptors in the regulation of pain, its effect in the spinal cord should be considered. It was reported that intrathecal administration of dopamine induced thermal antinociceptive effects through D2-like receptors when assessed by the tail flick test [346,347]. Activation of spinal dopamine D2-like receptors also reduced pain-related behavior following the establishment of inflammatory pain in both the affected and contralateral limb, while D2-like receptor antagonism decreased pain thresholds [348]. Intrathecal administration of dopamine also increases the mechanical nociceptive threshold as does quinpirole, whereas D1-like receptor activation had no effect [349]. Interestingly, in a recent study combining the dual 5-HT/NE reuptake inhibitor duloxetine with selective D1- or D2-like receptor agonists, it was demonstrated in the rat formalin test, that combination of all pharmacological agents produced superior analgesic effect than that obtained with duloxetine alone [350]. This potentiation of duloxetine-mediated antinociception is interesting because it suggestes that antidepressant that can simultaneously enhance serotonergic, noradrenergic and dopaminergic neurotransmission within nociceptive pathways should provide a broader spectrum of antinociception than dual reuptake inhibitors.

### 3.5. Triple Reuptake Inhibitors TRIs

Taken together, these pharmacological studies strongly suggest that drugs simultaneously inhibiting the re-uptake of 5-HT, NA and DA may provide a broader spectrum of pain relief in animal models of experimental pain than single- or dual-acting agents. Precedent for the use of TRIs in the treatment of clinical pain exists with nefopam, a non-narcotic analgesic marketed in Europe [351]. DA plays a critical role in nefopam analgesia as indicated by the observation that rats treated with 6-OHDA plus desipramine, which selectively depletes brain dopamine, have a marked reduction in nefopam-induced analgesia [352]. A specific role for D2-like receptors in nefopam-induced analgesia was demonstrated from the observation that the D2-like receptor antagonist sulpiride inbibited the behovorial response of nefopam [353]. The potential interest of TRIs in the relief of pain has been corroborated by a recent publication characterizing the antinociceptive effects of the TRI bicifadine in acute, persistent and chronic models of pain [320] (Table 3). In this study, bicifadine potently suppressed pain responses in two models of acute inflammatory pain in both rats and mice. It also normalized the nociceptive threshold in the complete Freund's adjuvant model of persistent inflammatory pain and suppressed mechanical and thermal hyperalgesia and mechanical allodynia in the spinal nerve ligation model of chronic neuropathic pain. Mechanical hyperalgesia was also reduced by bicifadine in the STZ model of neuropathic pain [320]. The impact of bicifadine on 5-HT, NE and DA neurotransmissions was confirmed by *in vitro* binding assays and intracerebral *in vivo* microdialysis study in freely moving rats. In a second study, another TRI, NS7051, has shown comparable antinociceptive properties to tramadol confirming the interest of these antidepressants in the relief of pain [354]. The molecule has undergone several Phase II and III trials for the treatment of pain, including acute postsurgical pain and chronic low back pain [355,356], and is being evaluated for painful diabetic neuropathy [357]. However, bicifadine has failed to meet endpoints in a number of trials such as diabetic neuropathy [358] suggesting that TRIs may be used in specific pain. Other TRIs currently under development for depression should draw attention for future investigation in the field of pain and confirm whether or not they display any activity in diabetic neuropathy.

### 3.5. Bridging the gap between preclinical and clinical studies

In the perspective to develop analgesic drugs, it is really important to take into consideration that pain research still faces enormous challenges and there remain many obstacles in the treatment of clinical pain. Unfortunately, there are celebrated examples of failed “translation,” where efficacy in animal models predicted efficacy in human clinical trials, but no efficacy was found. The most definitive example of this failure to translate is the substance P (SP) neurokinin-1 (NK1) antagonist MK-869, which failed despite demonstrations of adequate exposure, penetration, and occupancy [359]. There are several criticisms on animal paradigms that can account for the poor relationship between clinical and preclinical studies. First of all, animal pain models only produce conditions of tissue damage but not necessarily all dimensions of clinical pain. Even if animal models do duplicate clinical pain experience like humans, there is a lack of effective assessment tools to detect different dimension of pain experiences in animals [360]. In particular, evidence suggests that there is a network of brain regions involved in sensory, emotional, cognitive and motor processing. Combined to varying extents, and dependent upon conditions, these regions interact to generate the unique forms of pain experienced by different individuals [20]. Consequently, behavioral measures, although very informative, reduce significantly these dimensions of pain suggesting that an integrative approach is necessary to predict a clinical condition or the effects of an analgesic drug [360,361]. Secondly, while the prevalent method of pain assessment in clinical research is a self-reporting system using the visual analog (or numerical) pain scale, animals cannot tell us how much pain they are suffering. Thus, the probability of misinterpreting the evidence obtained from experiments in species other than humans is always present [362]. To overcome this issue, a new index for measuring pain in mice based on the rodents' facial expressions has been recently developed [363]. This study provides a new preclinical approach for scoring the therapeutic potential of analgesic drugs in complement of conventional methods such as the tracking of animal position, locomotion and gross behavior (e.g., grooming, drinking, eating, social approach). This index of pain relies on changes to facial characteristics being identical to those observed in humans. Thirdly, despite this advance in translational research, simple reflex tests (licking an inflamed paw or flicking the tail in response to heat) are still extensively used to measure pain in animal. However, such responses to evoke pain do not closely match the experience of continuous spontaneous pain in humans [364], especially because a persistent pain almost never results from heat stimulation in the clinical setting. In addition, reflexes tend to rely on the spinal cord and brain stem, whereas learned responses involve the brain's cerebral cortex (and are thus closer to the human condition). In these conditions, it seems that brain imaging would offer interesting opportunities to better understand the neurobiology of pain and evaluate the therapeutic potential of analgesic drugs [65]. Functional imaging may be used as a language of translation to help overcome some of these deficits. Others criticisms have been raised to explain the bridge between basic and clinical research in the field of pain. For example, it has been proposed that the existing models using inflammatory mediators such as formalin, carrageenan and Freund's adjuvant, are too artificial, [365]. It has also been argued that design issues and reporting standards in animal experiments are greatly inferior to those currently prevailing in human clinical trials [366]. Specifically, details regarding blinding, randomization, and data dropouts are relatively reported in animal studies of pain, likely leading to high experimental bias. Despite all these limitations that may explain, at least in part, the fact that several drugs active in animals study failed to become analgesic drugs in human, animal models have obvious advantages with respect to standardization of genetic and environmental backgrounds. Novel integrative approaches may help improve preclinical approach to nociception and also to enable the building of bridges between scientists and clinicians.

## 4. Conclusions and Clinical Perspectives

This review emphasized the fact that pain and depression share common neurotransmitters pathways and that monoaminergic reuptake inhibitors may relieve chronic pain. If chronic pain may result in depression, pain is also a symptom frequently observed in depressed patients [367]. If it is well established that brain 5-HT and NE systems play an important role in the inhibitory descending pathway controlling pain sensitivity, growing evidence suggests that DA may also have a strong influence at the spinal and supraspinal levels [341]. This supports promising efficacity of triple reuptake inhibitors in the treatment of chronic pain, which simultaneously enhance 5-HT, NE and DA neurotransmissions. Although the efficacy of SSRIs in the chronic pain management indicate that SSRIs are more effective than placebo [368], there are indications that the role of NE is more important than that of 5-HT in the relief of pain in preclinical but also in clinical studies [369]. Indeed, in humans, various neuropathic pain syndromes respond to dual-acting agents but not to SSRIs [370]. For example, venlafaxine was shown to be effective in several chronic neuropathic pain syndromes, whereas SSRIs such as fluoxetine or citalopram, did not show any activity [371,372]. In an extensive review of the clinical data, Fishbain and colleagues determined that, overall, dual acting antidepressants were more active than NRIs, which were more active than SSRIs [373]. A pooled analysis of 31 double-blind studies comparing venlafaxine with SSRIs found that the SNRI was significantly more effective than SSRIs in treating somatic symptoms associated with depression [374]. In particular, the proportion of patients with full remission of their somatic symptoms was significantly greater with venlafaxine than with an SSRI [374,375]. Recently, the SNRI duloxetine was the first reuptake inhibitor approved for the treatment of diabetic peripheral neuropathic pain [370,371]. Efficacy of duloxetine, venlafaxine and milnacipran was studied only in fibromyalgia. Six trials including 2220 participants involved duloxetine. Three studies included participants with painful diabetic neuropathy and three treated participants with fibromyalgia. In an open-label trial, duloxetine has shown consistent efficacy in painful diabetic peripheral neuropathy [376], with effectiveness sustained for one year [373]. Duloxetine (60 mg daily) was effective in treating painful diabetic peripheral neuropathy in the short-term to 12 weeks, in fibromyalgia over 12 weeks and 28 weeks. There is moderately strong evidence that duloxetine (60 mg and 120 mg daily) is efficacious for treating pain in diabetic peripheral neuropathy and fibromyalgia. Direct comparisons of duloxetine with other antidepressants and with other drugs whose efficacy was already demonstrated in neuropathic pain would be appropriate [377]. Venlafaxine has shown efficacy in polyneuropathies of different origins, but not in post-herpetic neuropathy [376]. Two case reports have shown bupropion effectiveness in the treatment of non-depressed patients with chronic low back pain of undetermined origin [378]. Furthermore, an open-label trial of 22 patients suggested that bupropion might be an effective and well-tolerated treatment of the neuropathic pain [379]. A randomized, controlled, double blind, crossover study has shown relief of pain in a group of patients with mixed aetiology of neuropathies [380]. The authors concluded that bupropion may be useful for treatment of neuropathic pain, but large-scale studies are needed to confirm this expectation. Regarding triple reuptake inhibitors, bicifadine is being tested in the treatment of chronic diabetic pain (phase II) and chronic low back pain (phase III). The results of these clinical trials should be available in the next months and will likely help determine whether the addition of the dopaminergic component to SNRIs can improve their analgesic efficacy.

To date, when comparative studies are available, clinical trials for pain invariably indicate a superiority of the dual-acting agents for pain treatment compared to single reuptake inhibitors. With the development of milnacipran, duloxetine and now the triple reuptake inhibitors such as bicifadine focusing in their ability to relieve pain, more extensive comparative data should be forthcoming to make a more evidence-based judgement on the superiority of the SNRIs and TRIs in these indications. In this context, preclinical studies are useful to unveil which antidepressants may display the best therapeutic profile and may determine whether doses that produce antidepressant effects also affect pain and reciprocally.

One question that also should draw our attention is the management of side effects and particularly whether the simultaneous enhancement of serotonergic, noradrenergic and/or dopaminergic neurotransmission mitigate or accentuate these side effects. Despite the much improved side effect profile of newer antidepressants, all of them are still associated with adverse effects that vary between classes and within each class [381]. SSRIs have replaced tricyclics as the drugs of choice in the treatment of depression, mainly because of their improved tolerability and safety. Common side effects of all SSRIs include transient nausea, diarrhea or constipation, dry mouth, insomnia, anxiety, somnolence, weight gain and sexual dysfuntion [381,382]. The dual acting antidepressants (SNRIs) have tolerability profiles that are comparable to those of the SSRIs however with an elevation in diastolic blood pressure particularly for venlafaxine [381,382]. Regarding antidepressants that increase DA neurotransmission, bupropion causes dry mouth, constipation, headache, nausea, excessive sweating and tremor but few cardiovascular effects and little sedation. In additon, it has shown a favorable profile in terms of both weight gain and sexual dysfunction [381,382]. As expected from these observations, data from various studies suggested that sexual dysfunction complaints particularly with the SSRIs and SNRIs can be managed by adding of dopaminergic antidepressant such as bupropion [383-385]. On the contrary, if certain medications do have weight gain as a side effect, there is a logical risk of severe increased weight gain when two of these antidepressants are combined [386]. Interestingly, in an effort to manage most SSRI-induced weight gain, it was reported that clinicians opt to switch agents rather than add a specific medication to the exisiting SSRI [387].

Regarding TRIs, a concern with drugs that block DA transporters is their potential reinforcing property and abuse liability [388]. Nevertheless, drugs that block DAT do not necessarily lead to dependence. Indeed, Volkow and collaborators showed that DA-transporter-blocking drugs must induce more than 50% DAT blockade to produce reinforcing effects [388]. Hence, DA reuptake inhibitors have been classified into two groups: type 1 blockers, which produce addiction and euphoria, and type 2 blockers, which do not [389]. It is thus possible that the capacity of DA reuptake blockers to produce dependence may involve other mechanisms that should carefully be considered with multi-targets agents such as triple reuptake inhibitors. Several Phase 1 studies have been conducted to evaluate their adverse effects. In a dose-escalating, placebo-controlled, double blind, Phase 1a trial, no adverse effects were observed after doses of DOV216303 several times higher than the projected therapeutic doses [390]. In a Phase 1b, clinical trial, 10 subjects were given either placebo (n = 3) or drug (n = 7) at 3 doses (25 mg b.i.d., 25 mg t.i.d. and 50 mg b.i.d.) for 10 days. No severe side effects were noted, although diarrhea, vomiting and nausea were observed. These observations contrast with those reported in a recent study showing that more patients suffering from Parkinson's disease and treated with the triple reuptake inhibitor tesofensine (82%) than in the placebo group (74%) experienced adverse events such as a higher rate of nervous system disorders (dyskinesia and headache), gastrointestinal tract disorders (nausea and constipation), and psychiatric disorders (halluci- nations and insomnia) [391]. Finally, weight loss has been observed as an adverse event in studies with tesofensine [392], prompting further research for the indication of obesity.

In conclusion, tolerability profiles of the antidepressant or combination of antidepressants should be considered when making treatment choices. The decision to use such combinations must be tempered by and weighed in conjunction with the knowledge that specific side effects are expected. It seems that the lack of research studies on the role of adjunctive treatments in the management of antidepressant-induced side effects favors monotherapy over polytherapies [387]. In this prospect, further efforts to evaluate the safety, efficacy, and place for antidepressant medication combinations are called for.

## Figures and Tables

**Table 1:** *In vitro* reuptake inhibition of monoamines transporters by monoaminergic reuptake inhibitors from rat synaptosomal fractions. * Ki values (nM).

**Drug**	**Mechanism**	***In vitro* functional activity – Uptake inhibition into rat brain synaptosomes (IC50, nM)**			**Ref**

		[3H]5-HT	[3H]NE	[3H]DA	
Citalopram	SERT	∼3.9	∼6100	>10,000	[140]
Escitalopram		∼2	∼2500	>10,000	[140]
Fluoxetine		∼32	∼630	∼5170	[141]
Fluvoxamine		∼10	∼1000	>10,000	[141]
Paroxetine		∼0.5	∼97	∼1170	[141]
Sertraline		∼58	∼1200	∼1100	[142]
Atomoxetine	NET	∼170	∼0.25	ND	[143]
Desipramine		∼380	∼1	∼3580	[141]
Reboxetine		∼794	∼2	>10,000	[144]
Duloxetine	SERT - NET	∼1.5	∼40	ND	[143]
Milnacipran		∼28	∼29	>10,000	[141]
Venlafaxine		∼63	∼316	∼7940	[144]
Bupropion	NET - DAT	∼19,900	∼1500	∼600	[145]
Nomifensine		∼830	∼6.6	∼48	[145]
DOV102677	SERT - NET - DAT	∼133	∼103	∼129	[146]
DOV21947		∼12.3	∼22.8	∼96	[147]
JNJ-7925476		∼1*	∼0.9*	∼2.6*	[148]
PRC200-SS		∼2.1*	∼1.5*	∼61*	[149]

**Table 2:** *In vivo* elecrophysiological effects of monoaminergic reuptake inhibitors on the firing activity of DR 5-HT, LC NE and VTA DA neurons in rats. [---]: 100% of inhibition, [--] between 50 and 100% of inhibition, [-] <50% inhibition, (0) No significant effect, (ND): Not Determined. Values into brakets indicate the dose that produces 50% of complete inhibition of neuronal activity (IC_50_).

**Drug**	**Mechanism**	***In vivo* functional activity – Inhibition of neuronal activity (IC_50_, μg/kg; iv)**			**Ref**

		**DR 5-HT**	**LC NE**	**VTA DA**	
Citalopram	SERT	- - - (∼250)	0	-	[116] [133] [162]
Escitalopram		--- (∼60)	0	0	[116] [133] [163]
Fluoxetine		--- (∼5000)	-	-	[162] [132]
Fluvoxamine		--- (∼600)	ND	-	[164]
Paroxetine		--- (∼240)	0	-	[164] [165]
Sertraline		--- (∼180)	ND	-	[164]
Atomoxetine	NET	ND	ND	ND	
Desipramine		0	--- (∼240)	ND	[166] [165]
Reboxetine		0	--- (∼110–480)	0	[134] [144] [158]
Duloxetine	SERT - NET	--- (∼10-700)	--- (∼480)	ND	[167] [168]
Milnacipran		--- (∼5700)	0	ND	[169]
Venlafaxine		--- (∼110-230)	--- (∼700-1000)	0	[165] [144]
Bupropion	NET - DAT	0	0	0	[170] [171]
Nomifensine		0	--- (∼40)	--- (∼450)	[172]
SEP225289	SERT - NET - DAT	-	- -	-	[173]
DOV216303		-	- -	-	[173]

**Table 3:** Effects of monoaminergic reuptake inhibitors on different models of acute and chronic pain in rodents.

**Drug (Mechanism)**	**Animal**	**Treatment (Duration; dose; route)**	**Pain Model**	**Test**	**Effect**	**Ref**
Citalopram (SERT)	Mice	A; 50 mg/kg; i.p.	Acute	Hot plate	+	[225]
	Mice	A; 20 mg/kg; i.p	Acute	Tail flick	−	[246]
				Hot plate test	−	
	Mice	A; 20 mg/kg; i.p.	Acute Inflammatory *PBQ*	Hot plate	++	[219]
				Abdominal writhes	+	
	Rat	A; 5 to 20 mg/kg; i.p.	acute	Hot plate	0	[222]
		A; 20 mg/kg; i.p.	Inflammatory *Formalin*	Formalin test	+	
	Rat	A; 3–30 mg/kg; i.p.	Acute	Tail flick	0	[247]
				Hot plate	0	
		A; 30 mg/kg; i.p.	Inflammatory *Formalin*	Formalin test	+	
		A; 3–30 mg/kg; i.p.	NP *CCI*	Von frey	0	
				Hind pressure	0	
		A; 10-30 mg/kg; i.p.	NP *CCI*	Radiant Heat	++	
	Rat	A; 3–10 mg/kg; i.p.	Visceral	Colonic distension	0	[248]
	Rat	C; 3–10 mg/kg; i.p.	Visceral	Colonic distension	0	
	Rat	C; 10 mg/kg.j; i.p.	Comorbid anxiety	Hargreaves	+	[249]
	Rat	- ; -; i.t.	Inflammatory *Acetic acid*	Abdominal writhing	0	[223]
	Rat	- ; - ; i.c.v	Inflammatory *Acetic acid*	Abdominal writhing	++	
	Rat	A; 10–25 mg/kg; i.p.	Acute	Tail flick	0	[224]
				Tail withdrawal	++	
		C; 10–25 mg/kg; i.p.	Acute	Tail flick	0	
				Tail withdrawal	0	
		A; 5–10 mg/kg; po	Inflammatory *Acetic acid*	Abdominal writhing	0	
		C; 25 mg/kg; po	Inflammatory *Acetic acid*	Abdominal writhing	0	
		A; 10–25 mg/kg; i.p.	Electrical	Flinch-jump test	0	
		C; 10–25 mg/kg; i.p.	Electrical	Flinch-jump test	0	
Escitalopram (SERT)	Mice	A; 0,5 to 50 mg/kg; i.p.	Acute	Hot plate	0	[225]
Fluoxetine (SERT)	Mice	A; 10–30 mg/kg; Po	NP *CCI*	Von Frey	++	[251]
	Mice	A; 10–40 mg/kg; Po	Acute	Hot plate	++	[252]
		C; 10 mg/kg; Po	Acute	Hot plate	++	
		A; 30 mg/kg; po	Inflammatory *Formalin*	Formalin test	++	
	Mice	A; 20 mg/kg; i.p.	Acute	Tail flick	++	[253]
			Inflammatory *Formalin*	Formalin test	++	
			Inflammatory *Carrageenan*	Von Frey	+	
	Mice	A; 15 μg; i.c.v.	NP *CCI*	Von Frey	0	[234]
				Tail Pinch	0	
		A; 5–10 mg/kg; i.t.	NP *CCI*	Von Frey	0	
				Tail Pinch	0	
		A; 15 μg; i.c.v.	NP *STZ*	Von Frey	0	
				Tail Pinch	0	
		A; 5–10 mg/kg; i.t.	NP *STZ*	Von Frey	0	
				Tail Pinch	0	
	Mice	A; 5–10–20 mg/kg; i.p.	acute	Hot plate	+	[226]
	Mice	A; 25 mg/kg; i.p.	Acute	Hot plate	+	[225]
	Mice	A; 10–20 mg/kg; i.p.	NP *STZ*	Tail Immersion	+	[254]
				Hot Plate	+	
	Mice	-; -; -	Acute	Hot plate	+	[255]
			NP *STZ*	Hot plate	0	
	Mice	A; 30 mg/kg; s.c.	Inflammatory *PPQ*	Abdominal writhes	+	[256]
	Rat	A; 56 mg/kg; s.c.	NP *SNL*	Von Frey	+	
	Rat	-; -; -	Acute	Tail flick	0	[238]
				Hot plate	0	
				Paw pressure	0	
	Rat	SC; 20 mg/kg.j; i.p.	NP *STZ*	Paw pressure	+	[257]
	Rat	-; -; s.c.	NP *CCI*	Radiant heat	0	[258]
	Rat	A; 3–10–30 mg/kg; s.c.	NP *SNL*	Von Frey	+	[259]
	Rat	A; 10–60 mg/kg; i.p.	Inflammatory *Carrageenan*	Paw oedema	++	[260]
		C; 20 mg/kg.j; i.p.	Inflammatory *Carrageenan*	Paw oedema	++	
	Rat	A; 10 mg/kg; i.p.	Inflammatory *Carrageenan*	Paw oedema	+	[261]
	Rat	C; 0,8 mg/kg; Po	Inflammatory *Formalin*	Formalin test	++	[233]
		A; 0.32 mg/kg; i.p.	Inflammatory *Formalin*	Formalin test	++	
		C; 0.16 mg/kg; i.p.	Inflammatory *Formalin*	Formalin test	++	
		A; 10 μg; i.t.	Inflammatory *Formalin*	Formalin test	++	
	Rat	A; 20 mg/kg; i.p.	Acute	Tail flick	++	[227]
				Hot plate	++	
		A; 5–20 mg/kg; i.p.	Inflammatory *Acetic acid*	Abdominal writhing	++	
		A; 1 μg; i.c.v.	Inflammatory *Acetic acid*	Abdominal writhing	++	
	Rat	A; 100–300 nmol; i.pl.	Inflammatory *Formalin*	Formalin 2nd phase	+	[262]
			NP *SNL*	Thermal hyperalgesia	0	
	Rat	A; 0.5–1–2–4 mg/kg; i.v.	Acute	Hot plate	0	[263]
				Tail flick	0	
		A; 0.25 mg/kg; i.v.	Electrical	NIWR	-	
	Rat	A; 3–30 mg/kg; s.c.	Inflammatory *Formalin*	Formalin test	0	[264]
			NP *SNL*	Von Frey	0	
				Pin Prick	0	
	Rat	A; 3–30 mg/kg; i.p.	Acute	Tail flick	+	[265]
		A; 30 mg/kg; i.p.	Inflammatory *Formalin*	Formalin test	+	
		A; 3–30 mg/kg; i.p.	NP *CCI*	Von Frey	++	
				Radiant heat	0	
			NP *SNL*	Von Frey	0	
				Radiant Heat	0	
	Rat	A; 5–10 mg/kg; -	acute	Tail jerk	0	[239]
Fluvoxamine (SERT)	Mice	A; 2–6–12 μg; i.c.v.	Inflammatory *Formalin*	Formalin test	++	[266]
			NP *SNL*	Radiant heat	++	
	Mice	A; 10 mg/kg; s.c.	NP *SNL*	Von Frey	+	[267]
				Hargreaves	0	
	Mice	A; 10–30 mg/kg; i.p.	Acute	Paw pressure	++	[268]
		A; 10–30–100 μg; i.t.	NP *SNL*	Von Frey	++	
		A; 10–30–100 μg; i.c.v.	NP *SNL*	Von Frey	0	
	Mice	A; 1–70 mg/kg;i.p.	acute	Hot plate	+++	[225]
	Mice	A; 30 mg/kg; i.p.	acute	Hot plate	+++	[269]
		A; 2.6–10 μg; i.t.	acute	Hot plate	++	
		A; 0.6–2.7 μg; i.c.v.	acute	Hot plate	++	
	Mice	A; 20 mg/kg; i.p.	Acute	Hot plate	++	[219]
			Inflammatory *PBQ*	Abdominal writhes	+	
	Rat	C; 20 mg/kg.j; i.p.	Arthritis	Paw pressure	0	[236]
	Rat	A; 20 mg/kg; i.p.	Inflammatory *Formalin*	Formalin test	+	[228]
	Rat	A; 0.5–1–2–4 mg/kg; i.v.	Acute	Hot plate	0	[263]
				Tail flick	0	
		A; 0.25 mg/kg; i.v.	Electrical	NIWR	−	
	Rat	A; 5 to 20 mg/kg; i.p.	acute	Hot plate	0	[222]
		A; 20 mg/kg; i.p.	Inflammatory *Formalin*	Formalin test	+	
	Rat	A; 1 mol to 1 μmol; i.t.	NP *CCI*	Von Frey	0	[241]
			NP *STZ*	Von Frey	0	
Paroxetine (SERT)	Mice	A; 4 mg/kg; s.c.	NP *SNL*	Von Frey	++	[102]
				Radiant Heat	++	
		SC; 5 mmol/j; i.c.v.	NP *SNL*	Von Frey	0	
				Radiant Heat	0	
	Mice	A; 5–10 mg/kg; i.p.	Acute	Hot plate test	++	[231]
	Mice	-; -; -	Acute	Hot plate test	++	[255]
			NP *STZ*	Hot plate test	0	
	Mice	A; 5–10–20 mg/kg; i.p.	Inflammatory *Acetic acid*	Abdominal writhing	++	[235]
	Mice	A; 5 mg/kg; i.p.	Acute	Hot plate test	++	[230]
	Mice	A; 3.8 mg/kg (*ED_50_*); s.c.	Inflammatory *Acetic acid*	Abdominal writhing	++	[270]
	Mice	A; 30 mg/kg; s.c.	Inflammatory *PPQ*	Abdominal writhes	+	[256]
	Rat	A; 10–30 mg/kg; s.c.	NP *SNL*	Von Frey	++	
	Rat	A; 0.1–100 nmol; i.t.	NP *STZ*	Von Frey	+	[241]
			NP *CCI*	Von Frey	0	
	Rat	A; 10–100 μg; i.t.	NP *SNL*	Von Frey	0	[271]
Sertraline (SERT)	Mice	A; 5 mg/kg; i.p.	acute	Hot plate	++	[232]
		C; 5 mg/kg; i.p.	acute	Hot plate	++	
	Rat	C; 30 mg/kg; Po	NP *STZ*	Hot plate	++	[229]
	Rat	A; 5–20 mg/kg; i.p.	acute	Hot plate	0	[222]
		A; 20 mg/kg; i.p.	Inflammatory *Formalin*	Formalin test	+	
	Rat	A; 3.6 to 28.8 mg/kg; i.p.	Inflammatory *Carrageenan*	Paw oedema	++	[261]
	Rat	A; 30 μg; i.t.	Acute	Tail flick	0	[242]
Desipramine (NET)	Mice	C; 10 mg/kg.j; i.p.	NP *SNL*	Von Frey	++	[272]
	Mice	A; 20 mg/kg; i.p.	acute	Tail flick	++	[273]
		A; 2.5 to 20mg/kg; i.p.	acute	Hot Plate	0	
		A; 2.5–20 mg/kg; i.p.	Inflammatory *Acetic acid*	Abdominal writhes	++	
		A; 2.5–20 mg/kg; i.p.	Inflammatory *Formalin*	Formalin	++	
	Mice	A; 20 mg/kg; i.p.	acute	Hot plate	+	[219]
			Inflammatory *PBQ*	Abdominal writhes	++	
	Mice	A; 30 mg/kg; s.c.	Inflammatory *PPQ*	Abdominal writhes	++	[256]
	Rat	A; 100mg/kg; s.c.	NP *SNL*	Von Frey	++	
	Rat	C; 10 mg/kg.j; i.p.	NP *SNL*	Von Frey	++	[274]
	Rat	A; 3–30 μg ; i.p.	Inflammatory *Formalin*	Formalin test	++	[275]
		A; 60–100 μg; i.t.	Inflammatory *Formalin*	Formalin test	++	
	Rat	A; 20 mg/kg; i.p.	acute	Hot plate	++	[222]
			Inflammatory *Formalin*	Formalin test	+++	
	Rat	A; 10–30–60–100 μg; it.	Inflammatory *Carrageenan*	Radiant heat	++	[276]
	Rat	A; 100–300 mmol; i.pl	Inflammatory *Formalin*	Formalin test	+	[262]
			NP *SNL*	Thermal hyperalgesia	++	
	Rat	A; 3μg; i.t.	Acute	Tail flick	++	[277]
	Rat	A; 25 mg/kg; -	Acute	Hot plate	0	[278]
	Rat	A; 3–10–30–100 mg/kg; s.c.	Inflammatory *Formalin*	Formalin test	++	[264]
		A; 10–100 mg/kg; s.c.	NP *SNL*	Von Frey	0	
				Pin Prick	++	
	Rat	A; 2 mg/kg; i.v.	NP *SNL*	Paw pressure	++	[279]
				«Pain related behaviour»	++	
	Rat	A; 25 mg/kg; Po	Acute	Tail flick	+	[280] [281]
	Rat	C; - ; Po	Acute	Tail flick	+	[282]
				Hot Plate	+	
Reboxetine (NET)	Mice	A; 10 mg/kg; i.p.	Acute	Hot plate	+	[283]
	Mice	C; 1.6 mg/kg.j; i.p.	NP *SNL*	Von Frey	++	[272]
	Mice	A; 3–10–30 mg/kg; s.c.	Inflammatory *PPQ*	Abdominal writhes	+++	[256]
	Rat	A; 100 mg/kg; s.c.	NP *SNL*	Von Frey	++	
	Rat	A; 0.5–5 μg; i.t.		Paw incisional injury	+	[284]
	Rat	A; 3–30 mg/kg; i.p.	Acute	Tail flick	+	[265]
			Inflammatory *Formalin*	Formalin test	++	
			NP *CCI*	Von Frey	0	
				Radiant heat	++	
			NP *SNL*	Von Frey	0	
				Radiant Heat	++	
Duloxetine (SERT – NET)	Mice	A; 30 mg/kg; i.p.	Acute	Tail flick	+	[253]
			Inflammatory *Formalin*	Formalin test	++	
			Inflammatory *Carrageenan*	Von Frey	+++	
	Mice	A; 5–10–20 mg/kg; i.p.	NP *STZ*	Tail immersion	++	[285]
				Hot plate	++	
	Mice	A; 30–100 mg/kg; po	NP *SNL*	Von Frey	+	[286]
	Mice	A; 3–30 mg/kg; i.p.	Chronic pelvic-perineal	“pain behaviour”	+	[287]
	Mice	A; 1–100 mg/kg; Po	Inflammatory *Acetic acid*	Abdominal writhing	++	[288]
			Inflammatory *Carrageenan*	Von Frey	++	
				Hargreaves	++	
	Mice	A; 1–30 mg/kg; i.p.	Acute	Tail Flick	0	[289]
				Hot Plate	++	
		A; 1 to 30 mg/kg; Po	Inflammatory *Acetic acid*	Abdominal writhing	+	
	Rat	A; 1–30 mg/kg; i.p	Inflammatory *Carrageenan*	Von Frey	++	
				Tail flick	++	
		A; 30 mg/kg; i.p.	Inflammatory *Capsaicin*	Von Frey	++	
	Rat	A; 0.16–40 mg/kg; i.p.	Inflammatory *Formalin*	Formalin test	++	[290]
		A; 5–10–20–40 mg/kg; i.p.	Stress induced	USV	++	
	Rat	A; 30–90–150 μg/kg; Po	Inflammatory *Osteoathritis*	Grip force	++	[291]
	Rat	A; 3-60 mg/kg; s.c.	Inflammatory *Formalin*	Formalin test	++	[292]
	Rat	A; 3–30 mg/kg; i.p.	Acute	Tail flick	0	[247]
				Hot plate	+	
		A; 30 mg/kg; i.p.	Inflammatory *Formalin*	Formalin test	++	
			NP *CCI*	Von frey	0	
				Hind pressure	++	
				Radiant Heat	++	
	Rat	A; 3–30 mg/kg; po	Acute	Tail flick	++	[293]
		A; 3–15 mg/kg; i.p.	Inflammatory *Formalin*	Formalin test	++	
		A; 5–30 mg/kg; po	NP *SNL*	Von Frey	+	
	Rat	A; 30 mg/kg; i.p.	EAE	Paint-Brush test	0	[294]
				Pinch test	0	
				Tail immersion	+	
				Cold plate	+	
	Rat	A; 0.4–20 mg/kg; i.p.	TASM ligation	Von Frey	++	[295]
Milnacipran (SERT – NET)	Mice	A; 210 ng–21 μg ; i.c.v.	NP *SNL*	Von frey	++	[296]
				Radiant heat	++	
		A; 210 ng–21 μg ; i.t.	NP *SNL*	Von frey	++	
				Radiant heat	++	
		A; 210 ng–21 μg ; local	NP *SNL*	Von frey	++	
				Radiant heat	++	
		A; 30–120 mg/kg; po	NP *SNL*	Von frey	++	
				Radiant heat	++	
	Mice	A; 2.5-20 mg/kg; i.p.	Inflammatory *Acetic acid*	Abdominal writhing	++	[297]
	Mice	A; 10 mg/kg; s.c.	NP *SNL*	Von Frey	++	[102]
				Radiant Heat	++	
	Rat	A; 1–30 μg; i.t.	Post operative	Von Frey	++	[298]
	Rat	A; 0.16–60 mg/kg; i.p	Inflammatory *Formalin*	Formalin test	+	[290]
		A; 2.5-20 mg/kg; i.p.	Stress induced	USV	++	
	Rat	C; 10 mg/kg.j; s.c.	NP *CCI*	Von Frey	++	[299]
	Rat	A; 60 mg/kg; i.p.	Acute	Analgesimeter	0	[300]
	Rat	A; 1μmol to 10mmol; i.t.	NP *CCI*	Von Frey	+	[241]
			NP *STZ*	Von Frey	+	
	Rat	A; 60 mg/kg; i.p.	NP *CCI*	Analgesimeter	++	[301]
	Rat	A; 3–100 μg; i.t.	NP *SNL*	Von Frey	++	[271]
	Rat	A; 10 mg/kg; i.v.	Visceral	Rectal distension	0	[302]
		A; 1 to 10 μg; i.t.	Visceral	Rectal distension	0	
	Rat	A; 10–30 mg/kg; i.p.	Inflammatory *Formalin*	Formalin test	+	[293]
		A; 300 mg/kg; po	NP SNL	Von Fre	+	
	Rat	A; 5 mg/kg; i.p.	Inflammatory *Formalin*	Formalin test	++	[228]
Venlafaxine (SERT –NET)	Mice	C; 10 mg/kg.j; i.p.	NP SNL	Von Frey	++	[272]
	Mice	-; -; -	Acute	Hot plate	+	[303]
	Mice	A; 70 mg/kg; i.p.	Acute	Hot plate	++	[304]
	Mice	A;l 1 to 30 mg/kg; i.p.	Acute	Hot plate	+	[305]
	Mice	A; 1–30 mg/kg; i.p.	Acute	Hot plate	++	[306]
	Rat	A; 10–30 mg/kg; i.p.	Inflammatory *Formalin*	Formalin test	++	[293]
		A; 300 mg/kg; po	NP *SNL*	Von Frey	+	
	Rat	A; 50–100 mg/kg; i.p.	Inflammatory *Carrageenan*	Von Frey	++	[307]
	Rat	A; 2.5–5–10 mg/kg; s.c.	NP *STZ*	Paw pressure	++	[272]
	Rat	A; 10–40 mg/kg; s.c.	NP *Vincristin*	Paw pressure	++	[308]
	Rat	A; 7.5 mg/kg; s.c.	NP *Oxaliplatine*	Tail-immersion test	++	[309]
	Rat	A; 22 mg/kg; i.p.	NP	Paw pressure	0	[310]
	Rat	A; 25 mg/kg; i.p.	Acute	Hot plate test	+++	[311]
	Rat	A; 10–25–50 mg/kg; s.c.	NP *SNL*	Von Frey	0	[312]
				Formalin test	++	
	Rat	A; 2.5 mg/kg; s.c.	Acute	Paw pressure	0	[313]
			Inflammatory *Formalin*	Formalin test 2nd phase	++	
	Rat	- ; - ; -	NP *SNL*	Paw pressure	++	[314]
	Rat	SC; 22 mg/kg ; Po	NP *CCI*	Radiant Heat	++	[315]
	Rat	A; 3–100 mg/kg; i.p.	Acute	Tail flick	+	[265]
			Inflammatory *Formalin*	Formalin test 2nd phase	+	
			NP *CCI*	Von Frey	0	
				Radiant heat	+	
			NP *SNL*	Von Frey	0	
				Radiant Heat	0	
Bupropion (NET – DAT)	Mice	A; 10–30 mg/kg; Po	NP *CCI*	Von Frey	++	[251]
	Rat	A; 3–30 mg/kg; i.p.	Acute	Tail flick	+	[265]
			Inflammatory *Formalin*	Formalin test	0	
			NP *CCI*	Von Frey	++	
				Radiant heat	+	
			NP *SNL*	Von Frey	++	
				Radiant Heat	0	
Nomifensine (NET – DAT)	Rat	A; 0.625–5 mg/kg; s.c.	Inflammatory *Formalin*	Formalin test	++	[316]
	Rat	A; 25 mg/kg; i.p.	Acute	Charpentier pain mode	0	[317]
	Rat	A; 0.1–10 mg/kg; i.p.	Acute	Tail immersion	-	[318]
	Rat	A; 10–40 mg/kg; s.c.	Acute	Hot plate	0	[319]
		A; 2.5–5–10 mg/kg; s.c.		Tail immersion	-	
Bicifadine (SERT – NET – DAT)	Rat	A; 4-8-12 mg/kg; po	Acute inflamatory	Randall Selito test	++	[320]
		A; 50 mg/kg; po		Kaolin-induced arthritis test	++	
		A; 25–50–75–100 mg/kg; po	Acute	Tail flick	++	
				Hot plate	0	
		A; 10–30 mg/kg; po	Inflammatory *PPQ*	Abdominal writhing	++	
		A; 5–10–20–40 mg/kg; po	Visceral	Colonic distension	++	
		A; 40–60 mg/kg; po	Inflammatory	Freund adjuvant	++	
		A; 10–20–30 mg/kg; po	Inflammatory *Formalin*	Formalin test	++	
		A; 40 mg/kg; po	NP *SNL*	Paw pressure	++	
		A; 40 mg/kg; po		Von Frey	++	
		A; 12.5–25–100 mg/kg; po		Radiant heat	++	
		A; 12.5–25 mg/kg; po	NP STZ	Paw pressure	++	

*Treatment* A: acute, SC: Subchronic from 2 to 5 consecutive days, C: Chronic more than 5 consecutive days. i.p.: intreperitoneal; i.c.v: intracerebroventricular; i.t.: intrathecal; i.v.intravenous; po: per os; s.c.: subcutaneous; *Pain model* NP: neuropathic pain; SNL: sciatic nerve ligation; CCI: chronic constriction injury; PH: post herpetic; EAE: experimental autoimmune encephalomyelitis; TASM: tendon of the anterior superficial part of the rat masseter muscle; NIWR: noxious-induced withdrawal reflexes; PBQ: *para*-benzoquinone; PPQ: *para*-phenylquinone. *Test* USV: Ultrasound vocalization. *Effect* (−) pronociceptive effect; (0) No effect; (++) antinociceptive effect; (+) when authors specifie “weak”, “little” or “mild” antinociceptive effect; (+++) when authors specifie “strong” or “important” antinociceptive effect.

## Abbreviations

ACC: anterior cingulate cortex
5-HT: 5-hydroxytryptamine or serotonin
5-HTT: serotonin transporter
CPu: caudate putamen
CRF: corticotropin-releasing factor
DA: dopamine
DAT: dopamine transporter
DRN: dorsal raphe nucleus
FC: frontal cortex
IC: insular cortex
LC: locus coeruleus
mPFC: medial prefrontal cortex
NAcc: nucleus accumbens
NE: norepinephrine
NET: norepinephrine transporter
PAG: periaqueductal grey
PFN: parafascicular nucleus
RVM: rostral ventromedial medulla
SN: substantia nigra
SNL: sciatic nerve ligature
SNRI: serotonin and norepinephrine reuptake inhibitor
SP: substance P
SSRI: serotonin selective reuptake inhibitor
STZ: streptozotocin
TRI: triple reuptake inhibitor
VTA: ventral tegmental area
